# Phenomenology, Epidemiology and Aetiology of Postpartum Psychosis: A Review

**DOI:** 10.3390/brainsci11010047

**Published:** 2021-01-04

**Authors:** Amy Perry, Katherine Gordon-Smith, Lisa Jones, Ian Jones

**Affiliations:** 1 Psychological Medicine, University of Worcester, Worcester WR2 6AJ, UK; k.gordon-smith@worc.ac.uk (K.G.-S.); lisa.jones@worc.ac.uk (L.J.); 2 National Centre for Mental Health, MRC Centre for Neuropsychiatric Genetics and Genomics, Cardiff University, Cardiff CF24 4HQ, UK; jonesir1@cardiff.ac.uk

**Keywords:** postpartum psychosis, aetiology, triggers

## Abstract

Postpartum psychoses are a severe form of postnatal mood disorders, affecting 1–2 in every 1000 deliveries. These episodes typically present as acute mania or depression with psychosis within the first few weeks of childbirth, which, as life-threatening psychiatric emergencies, can have a significant adverse impact on the mother, baby and wider family. The nosological status of postpartum psychosis remains contentious; however, evidence indicates most episodes to be manifestations of bipolar disorder and a vulnerability to a puerperal trigger. While childbirth appears to be a potent trigger of severe mood disorders, the precise mechanisms by which postpartum psychosis occurs are poorly understood. This review examines the current evidence with respect to potential aetiology and childbirth-related triggers of postpartum psychosis. Findings to date have implicated neurobiological factors, such as hormones, immunological dysregulation, circadian rhythm disruption and genetics, to be important in the pathogenesis of this disorder. Prediction models, informed by prospective cohort studies of high-risk women, are required to identify those at greatest risk of postpartum psychosis.

## 1. Introduction

Severe postpartum mood disorders are an important clinical and public health concern, being associated with high economic costs [1], increased risk of maternal suicide [2] and potential impact on developmental outcomes in the offspring [3]. Mood disturbances associated with childbirth are typically classified into three main groups, ranging from the mild and self-limiting “baby blues”, to more clinically significant and impairing episodes of postpartum depression and finally, postpartum (puerperal or postnatal) psychosis. While the baby blues and postpartum depression are both common in the general population [4,5], postpartum psychoses are less common, occurring following one to two in every 1000 deliveries [6]. These episodes represent some of the most severe forms of postpartum mood disorders, which, as psychiatric emergencies, can have devastating consequences for the mother, baby and wider family [7,8,9]. Understanding the mechanisms by which postpartum psychosis occurs is crucially important for risk prediction and effective management of these episodes. In this review, we focus on summarising the current evidence regarding the phenomenology, epidemiology and aetiology of postpartum psychosis.

## 2. Phenomenology

The term postpartum psychosis is traditionally used to describe severe episodes of mood disorder that have very sudden onset after childbirth, usually within the first two weeks [10,11,12]; see Table 1 for an overview. In many cases, postpartum psychosis is characterised by the onset of mania or a mixed mood episode, yet depression, lability of mood, perplexity (extreme confusion) and anxiety are also common [13,14,15,16]. This myriad of symptoms, combined with a mixed mood presentation, characteristically results in a ”kaleidoscopic” clinical picture that may also be interspersed with brief, symptom free periods of lucidity [17]. Psychotic symptoms are usually a co-occurring feature of the illness, estimated to be present in more than 70% of cases [16]. While a broad range of psychotic symptoms can occur (such as hallucinations and delusions of being controlled or misinterpretation), persecutory delusions and delusions of reference may be the most common [16]. There is some evidence to indicate that compared to similar mood episodes (of mania or psychosis) unrelated to childbirth, postpartum psychosis presents as atypical, with visual hallucinations and perplexity being reported more frequently in postpartum onset episodes [14,15], while conversely, grandiose delusions may be less likely to occur [14]. 

In cases in which delusional ideation is present, the content often relates to the infant [16,18]. This may lead to an increase in protective behaviours by the mother, or in some cases, of risk of neglect or abuse [18]. While evidence suggests homicidal thoughts are more likely to occur in postpartum psychosis compared to non-psychotic episodes of postpartum mood disorder [19,20], infanticide is rare, occurring in between 1 and 4.5% of all cases [9]. Women who experience postpartum psychosis also frequently report thoughts of self-harm [16], placing them at greater risk of suicide compared to mothers with psychiatric disorders that had onset at other times [21]. In the United Kingdom, suicide remains a leading cause of maternal death within the first postpartum year [2], further highlighting the importance of effective prevention and treatment of these episodes. 

## 3. Epidemiology

Compared to before or during pregnancy, the postpartum period has been demonstrated as a time of particularly high risk of severe psychiatric disorders, implicating childbirth in the triggering of these episodes [10,12]. In a seminal study conducted in a UK population sample, the risk of psychiatric admission for a psychotic or mood illness was 22 times greater in the first month following delivery compared to before pregnancy [10]. This risk was further increased among women who were primiparous (i.e., had given birth to their first baby), with psychiatric admissions being 35 times more likely. 

While more than 40% of women affected by postpartum psychosis have no history of severe psychiatric illness [12], the remainder present with a recurrence of a pre-existing psychiatric illness, predominantly of a psychotic or mood disorder [11,12]. Evidence robustly indicates a strong and specific relationship with bipolar disorder, suggesting that in most cases, postpartum psychosis may be a manifestation of bipolar disorder in women vulnerable to the puerperal trigger. A recent meta-analysis estimated that as many as one in five women with bipolar disorder are affected postnatally by a psychotic or manic episode [22], a rate considerable higher than that observed in the general population (1–2 in every 1000 deliveries) and other psychiatric disorders. Among women with schizophrenia or any other mood or psychiatric disorder, the risk of admission for psychotic or severe mood episodes was only marginally increased within the first three months following childbirth compared to a sample of non-mothers matched for psychiatric diagnosis (relative risk of 4.6 and 3.0, respectively) [23]. In contrast, the risk of psychiatric admission for a recurrence of bipolar disorder in the postpartum period is especially high, being 37 times more likely than in women who had never given birth [23]. This finding has since been replicated in a more recent study, showing psychiatric admissions within the first 6 weeks of childbirth to most commonly be for a severe recurrence of bipolar disorder (14.4%), while other psychiatric disorders accounted for a considerably lower proportion of all admissions (ranging from 1.4 to 7.2%) [12].

## 4. Link with Bipolar Disorder

The close relationship between postpartum psychosis and bipolar disorder is further suggested by findings from longitudinal research. Of women who experience the first onset of mood disorder in the postpartum period, the initial episode frequently marks the onset of bipolar disorder [24,25], particularly if the postpartum episode required admission to hospital or occurred within the first two weeks following childbirth [24]. First onset depression that occurs following delivery may also be indicative of an underlying bipolar illness. Compared to first onset depression outside of the postpartum period, atypical symptoms such as psychosis and mixed mood features have been shown to be more common in postnatal episodes [26]. In the same study, women with first onset postpartum depression were also more likely to report a family history of bipolar disorder compared to women whose first episode occurred unrelated to childbirth. 

## 5. Nosology

The nosological status of postpartum psychosis remains a contentious issue. While proposed by some to be a distinct disorder [27], the predominant view is that childbirth most commonly acts as a potent trigger of established mood disorders. This view is reflected in current operational diagnostic criteria. While ICD-10 does include the category ”mental or behavioural disorders associated with pregnancy, childbirth or the puerperium”, this diagnosis can only be applied to episodes occurring with onset within the first 6 weeks of delivery and only if these episodes cannot be classified elsewhere [28]. In accordance with DSM-5 criteria, episodes of postpartum psychosis are usually classified as severe episodes of mood illness, denoted with a peripartum specifier if onset occurs in pregnancy or within 4 weeks of delivery [29]. However, episodes with onset in the postpartum period are not distinguished from those occurring during pregnancy. 

The challenges of classifying postpartum psychosis according to DSM and ICD criteria has contributed to confusion about how these episodes should best be conceptualised. Currently, there is no universally accepted definition of postpartum psychosis. In some cases, the concept is limited strictly to mood disorders with psychotic features, while in others, it is expanded to include the less common occurrence of mania without psychosis that has onset following childbirth. Nonetheless, while the boundaries of this definition remain uncertain, the core of the concept is clear. In our review, we, therefore, focus on examining literature that has defined postpartum psychosis as severe episodes of mania (with or without psychotic features) or depression with psychosis that have onset soon after delivery. In contrast, other postpartum mood disorders, such as episodes of major depression without psychosis are not included.

## 6. Prognosis and Recurrence 

Following treatment, recovery from an initial episode of postpartum psychosis is excellent in most women [30,31]. However, of affected women who do go on to have further children, more than 50% are at risk of the recurrence of a perinatal mood episode [32]. Studies of risk of postpartum recurrence among women with a history of both bipolar disorder and postpartum psychosis are limited. However, in a large retrospective sample of parous women (women who had given birth at least once) with bipolar disorder, we recently reported as many as 43% women with bipolar I disorder and a perinatal history of mania or psychosis to be at risk of a severe recurrence within a subsequent pregnancy [33]. This is compared to approximately 10% women with bipolar I disorder and 2% of those with bipolar II disorder without such perinatal history. 

## 7. Psychotropic Medication Use

The high rate of recurrence of existing mood disorders in the postpartum period may, in part, be accounted for by medication factors. For example, women with bipolar disorder face difficult decisions regarding the use of psychotropic medication during the perinatal period, often in the absence of an established evidence base [34]. Many subsequently choose to withdraw medication due to fears of teratogenic effects or because they have an intention to breastfeed [34]. A meta-analysis (comprised predominantly of case-reports and small retrospective studies of lithium) has shown that among women who do withdraw prophylactic mood stabilising medications during pregnancy, risk of a postpartum recurrence of bipolar disorder is especially high, with 66% of women being affected. This compared to 23% of women who continued prophylactic mood stabilisers during pregnancy [22]. Nevertheless, a recent study has demonstrated risk of postpartum recurrence of bipolar disorder to remain high, despite prophylactic psychotropic medication use during pregnancy and the early postpartum period [35]. Together, this would suggest that factors other than medication are likely to play an important role in the aetiology and triggering of postpartum psychosis. This notion is further supported by one key study, in which recurrence of bipolar disorder was compared between groups of women who had withdrawn mood stabilising medication either during or outside of the perinatal period [36]. In this study, pregnant women who withdrew mood stabilising medication were no more likely to experience a recurrence of bipolar disorder compared to an age matched sample of non-pregnant women followed over the same time period. However, of women who were euthymic at the 40-week follow-up, recurrence between weeks 41 and 64 was 2.9 times more frequent among parous women in the postpartum period compared to women in the non-postpartum equivalent period.

## 8. Aetiological and Triggering Factors

Postpartum psychosis provides a unique opportunity to investigate the aetiology and potential triggering factors of psychotic and mood disorders. In no other psychiatric condition are we able to predict as precisely the onset of the disorder, definable to within such a narrow timeframe in relation to a biological trigger. Similar to psychiatric disorders more generally, the aetiology of postpartum psychosis is likely to be explained by a complex interaction of biological, psychological and social factors. In the remainder of this review, we examine whether the following psychosocial and neurobiological factors play a role in the onset of postpartum psychosis: (i) obstetric factors, (ii) psychological and social stressors, (iii) sensitivity to hormone changes that occur in relation to labour and parturition, iv) sleep and circadian rhythm disruption occurring within the perinatal period, (v) immunological factors and (vi) genetics.

### 8.1. Obstetric Factors

Several obstetric factors, such as mode of delivery [37,38], sex of the infant [39] and complications of pregnancy or delivery [25,38,40,41] have been investigated in relation to postpartum psychosis, yet results to date have mainly been inconsistent or without replication. In a recent population study of more than one million women, the stillbirth of an infant was associated with a 2.5 times increased risk of severe psychiatric disorder within the first postpartum year [42]. However, outcome in this study was broadly defined, encompassing a range of psychopathology such as suicidality, anxiety disorders and acute stress reactions. The specific association between stillbirth and narrowly defined postpartum psychosis was not examined. Of all obstetric factors investigated, only primiparity has been reliably associated with the onset of postpartum psychosis [37,43,44], a finding that does not appear to be explained by a bias of affected women being less likely to have additional children [44]. Nonetheless, the nature of this relationship remains unclear, and is potentially explained by significant changes in biological or psychosocial factors that, in particular, are associated with pregnancy and the birth of a first baby.

### 8.2. Psychological and Social Factors

Psychological and social factors have been shown to be important in the triggering of postpartum depression [45,46]; however, these factors appear to play less of a role in the triggering of postpartum psychosis. Personality traits, cognitive styles or affective temperaments show no specific relationship to postpartum psychosis [47], despite these factors being associated with postpartum depression and with bipolar disorder more generally. The potential relationship between life stressors (such as history of trauma) and the onset of postpartum psychosis has been examined in several studies, yet no consistent associations have been found with adverse life events occurring within pregnancy or the 12 months prior to delivery [48,49,50,51], nor with those occurring during childhood [46,52]. Of investigations of within-pregnancy psychosocial factors, findings are inconsistent, with some studies identifying the lack of a partner to be associated with postpartum psychosis [10,53,54], while others have not [49,55,56]. Though marital difficulties during the perinatal period may be related [48], it is not clear whether this factor may contribute to the onset of symptoms or be a consequence of the illness. Unmet maternal expectations in relation to childbirth and breastfeeding are associated with feelings of shame and failure and may increase risk of depressive illness and anxiety in the postpartum period [57,58]. While these factors require further examination in relation to postpartum psychosis, limited qualitative data in this area suggest some women perceive difficult birth experiences and unsupportive family relationships to be potential causes of their postpartum psychotic illness [59]. 

### 8.3. Hormonal Factors

The rapid onset of postpartum psychosis following parturition suggests that hormonal factors are potentially involved in the aetiology of this disorder. This hypothesis is plausible given that the abrupt withdrawal of progesterone and oestrogen that occurs postbirth [60] coincides with the peak timing of onset of postpartum psychosis. In line with this theory, associations have been reported between mood episodes triggered by childbirth, the premenstrual phase of the menstrual cycle and/or the menopause in women with bipolar disorder [57,58,59,60,61]. Notably, a proportion of women are also seemingly only vulnerable to experiencing severe mood episodes in relation to childbirth [29]. The mechanism by which hormonal factors increase vulnerability to postpartum psychosis is likely to extend beyond the role of progesterone and oestrogen alone, also involving interactions with other reproductive hormones and neurotransmitters. Both oestrogen and progesterone are known to interact with monoaminergic functioning, with oestrogen having an overall facilitatory effect on dopaminergic systems [61]. Given that monoamines are well evidenced to play a role in mood and psychotic disorders outside of the perinatal period [62,63,64], researchers have postulated that the precipitous drop in hormones following childbirth may result in dopaminergic hypersensitivity in some women vulnerable to the childbirth trigger [65]. There is also evidence to suggest that prolactin may be implicated in this potential mechanism. Prolactin inhibitors (known dopamine D2 receptor agonists) are frequently prescribed to treat medical illnesses such as hyperprolactinemia and Parkinson’s disease, and have been shown to induce or exacerbate mania and psychosis outside the postpartum period in some individuals with these disorders [66,67,68]. Interestingly, increased prolactin plasma levels have also been associated with an increased severity of psychotic symptoms in a sample of women with first episode psychosis, controlling for the potentially confounding effect of long-term antipsychotic use [69]. Studies examining the role of prolactin in the occurrence of postpartum psychosis are rare. However, based on a small collection of case-reports and case-series, a recent systematic review showed prolactin inhibitors, in particular Bromocriptine, to potentially increase risk of psychosis in the postnatal period when utilised to supress lactation in women with and without a history of psychiatric disorder [70]. 

Despite plausibility of the hormone withdrawal hypothesis, evidence in support of this theory appears largely circumstantial. Absolute levels of steroid hormones do not appear to differ between women affected by postpartum mood disorders and those who remain well [71]. For this reason, the assumption that the abrupt withdrawal of hormones is a direct causal factor in postpartum psychosis has been criticised for being overly simplistic [65]. Nonetheless, it remains possible that some women are differentially sensitive to normal endocrine changes that occur in relation to pregnancy and childbirth [72]. Evidence to support this is provided by a seminal study, in which the supraphysiologic levels of oestrogen and progesterone typically observed in pregnancy were simulated in a sample of women with and without a history of postpartum depression [71]. Following blind withdrawal of these steroids, 62% women with a history of postpartum depression developed symptoms of mood disorder, compared to none in the control group. However, such studies in relation to postpartum psychosis are currently lacking.

### 8.4. Sleep Deprivation/Circadian Rhythm Disruption

A further factor implicated in the triggering of postpartum psychosis is the potential disruption to circadian rhythms that occurs in relation to acute sleep loss during the perinatal period. This theory is primarily based on three lines of reasoning. The first is that sleep loss is both a symptom and frequently cited precipitant of mania unrelated to childbirth [73], while second, acute sleep disruption occurs almost universally in women during the immediate postpartum [74]. Third, neurotransmitters such as dopamine and serotonin are well evidenced to play a role in mood disorders, the systems of which may interact and overlap to also regulate circadian rhythms, including those relating to the sleep/wake cycle and hormone secretion [75,76]. 

Studies directly investigating the role of sleep loss in the triggering of postpartum psychosis are scarce. However, of those conducted in this area, findings have been promising. When compared to a matched parous control group, women with postpartum psychosis have been shown to experience a longer duration of labour and be more likely to give birth during the night-time, suggesting these women experienced increased and sustained sleep loss associated with labour [77]. Moreover, in a large sample of parous women with bipolar disorder who were assessed retrospectively, we have shown that those who reported sleep loss as a lifetime trigger of manic episodes were approximately twice as likely to have a history of postpartum psychosis compared to women who did not report this [78]. In contrast, a prospective study of ”high risk” women (with a history of bipolar disorder or postpartum psychosis) demonstrated sleep patterns to be similar during the perinatal period to those of a healthy control group of pregnant women [79]. Importantly, however, only three women in this study subsequently experienced the onset of postpartum psychosis; therefore, comparison of perinatal sleep patterns between women who did and did not experience postpartum psychosis was not possible. 

### 8.5. Immunological Factors

Evidence to indicate that risk of postpartum psychosis is increased with first pregnancy, combined with the fact that pregnancy itself is associated with considerable and highly modulated changes in immunological functioning [80], suggests that postpartum psychosis may, in part, be explained by dysfunction or dysregulation of immunological systems. Support for this hypothesis is provided by several lines of enquiry. First, immunological factors have been implicated in the aetiology of psychoses unrelated to childbirth [81] and specifically in bipolar disorder [82]. Inflammatory cytokines (responsible for cell signalling and promotion of systemic inflammatory response) are found to be significantly elevated during acute phases of bipolar-related mood episodes, most consistently during episodes of mania [83,84]. Second, immune disorders such as postpartum thyroiditis, rheumatoid arthritis and multiple sclerosis are typically exacerbated in the postpartum period, sharing similarities with the clinical course of postpartum psychosis [85]. Third, psychiatric symptoms resembling postpartum psychosis have been reported to occur in cases of immunological disorders such as anti-N-methyl-D-aspartate receptor encephalitis [86] and pre-eclampsia [87], a disorder also linked with first pregnancies [88]. Notably, however, while a recent meta-analysis has shown pre-eclampsia to be associated with an increased severity of depressive symptoms in the postnatal period, no such relationship was observed with postpartum psychosis [89].

Direct investigations of inflammatory cytokines suggest generalised immune dysregulation among women with postpartum psychosis. For example, alterations in cytokine levels of pro-inflammatory T-helper cells type 1 and 17 and regulatory T-cells have been observed in women with postpartum psychosis, when compared to healthy parous controls [90]. Abnormal levels of cytotoxic natural killer cells may also play a role [85,90]. Moreover, monocyte levels and upregulation of inflammatory monocyte genes have been reported in women admitted for first-onset postpartum psychosis, compared to postpartum and non-postpartum control groups who were unaffected [85]. Autoimmune thyroid dysfunction, which progresses significantly faster to clinical thyroid disease has also been more frequently observed in women admitted for postpartum psychosis compared to a healthy postpartum control group [91]. In this study, rates of thyroid dysfunction and of clinical thyroid disease remained significantly elevated at 9 months postpartum and were found to be independent of the effect of lithium treatment, which has been demonstrated to adversely affect thyroid function. 

### 8.6. Genetic Factors

Strong evidence also implicates genetic factors in the pathophysiology of postpartum psychosis. Mood disorders related and unrelated to childbirth have been shown to aggregate within families, implying a degree of shared genetic susceptibility to mood disorders in general. Evidence shows that as many as 40–50% of women with a history of postpartum psychosis screen positive for a family history of mood disorders among first- and second-degree relatives [92,93,94,95], a rate much higher than that observed in the general population. Specific vulnerability to the childbirth trigger of severe mood episodes may also be familial, with findings further suggesting postpartum psychosis may be a marker for a genetically heterogenous subtype of bipolar disorder. In one key study, 74% of women with bipolar disorder with a positive family history of postpartum psychosis in a first degree relative were found to have also experienced a severe mood episode triggered by childbirth, compared with only 30% of women with bipolar disorder without such family history [96]. 

Though genetic factors are suspected to play a role, specific genes determining risk of postpartum psychosis are yet to be identified. Nevertheless, progress in this area is being made. In a genomewide scan of 54 sibling pairs with bipolar disorder and at least one family member with a history of postpartum psychosis, significant linkage was identified at chromosomal location 16p13 and suggestive linkage at 8q24 [97]. These locations were not identified in relation to bipolar disorder more generally, suggesting specificity to the childbirth triggering of mood episodes. Several candidate genes have also been targeted for further investigation, with those involved in hormonal [98,99], neurotransmitter [100,101], immunological [102] and circadian rhythm [103] functioning receiving particular attention (see Table 2 for overview). One such gene, methyl transferase like 13, has been associated with postpartum mood symptoms in broadly defined mood disorders [104] and with postpartum psychosis in diagnostically heterogenous samples [98]. Variations in the 5-HTTT serotonin transporter gene, the expression of which is known to be influenced by hormones, has also been associated with postpartum psychosis among women with bipolar disorder [100]. Specifically, the presence of the STin2.12 allele was associated with a four times increased risk of postpartum recurrence. This effect size was further increased when analysis was restricted to only multiparous women (women who had given birth more than once) with a recurrent history of postpartum psychosis. These findings have since been replicated, with associations between a number of polymorphisms of serotonergic genes (including 5-HTTT) and postpartum psychosis being found in an independent sample of women with bipolar disorder [101].

Although several studies have reported positive associations with a number of candidate genes, to date, few of these findings have been replicated. Consistent with many other complex disorders, the likelihood of these individual studies representing true positives is low, demonstrating the need for genomewide association studies (GWAS) in this area. GWAS of postpartum psychosis are a promising area for future research, given that in contrast with immunological or hormonal factors, inherited genetic factors are stable over time and DNA samples can more feasibly be obtained. Findings of GWAS in relation to other severe psychiatric disorders such as bipolar disorder and schizophrenia demonstrate feasibility of this approach, identifying common genes that increase risk by relatively small amounts (single nucleotide polymorphisms) and rarer variants that increase risk more substantially (copy number variants) [105]. From these studies, it is, however, clear that much larger samples are required to achieve sufficient analytical power. To date, the rare occurrence of postpartum psychosis in the general population, combined with difficulties concerning the nosology has hampered efforts to obtain the sufficient sample size required. Nevertheless, international collaborative efforts are now underway. 

## 9. Conclusions

Childbirth is a potent trigger of episodes of severe mood disorder. While half of all women who experience postpartum psychosis do not have a history of psychiatric illness, those with bipolar disorder or a history of postpartum psychosis are at particularly high risk. In order to work with women to prevent episodes and ensure perinatal mental healthcare is focussed on those who can benefit the most, predictive models are required to identify women at greatest risk. Fortunately, the onset of postpartum psychosis is isolated to within a very specific timeframe, providing an opportunity to study the aetiology of these episodes in a way that is unique from other psychiatric illnesses. Further research in this area would provide an opportunity to understand the pathophysiology of not only postpartum psychiatric illnesses but also of psychiatric disorders more widely. This review has identified several areas where future research is needed (see Table 3 for summary). Ideally, work around the nosology of postpartum mood disorders will ensure these episodes can, in time, be more accurately coded and classified within operationalised diagnostic criteria, enhancing our ability to identify relevant cases for further research. Collaborative efforts should also continue to focus on building large samples of women with postpartum psychosis, so that genetic and other biomarkers can be identified. Finally, efforts should be placed on utilising a range of paradigms to examine other potential aetiological factors in large prospective cohorts of women at high risk.

## Figures and Tables

**Table 1:** Phenomenology and epidemiology of postpartum psychosis.

Phenomenology	Mania/mixed mood state Depression Anxiety Perplexity ”Kaleidoscopic” presentation Psychotic symptoms Mood lability
Prevalence	0.001–0.002% (1–2/1000)
Peak timing of postpartum onset	Days 1–14
Duration	Weeks to months

**Table 2:** Summary of literature identifying potential genetic risk markers for postpartum psychosis.

Author (Year)	Sample	Genetic Marker(s)	Finding
Jones et al. (2007) [97]	36 pedigrees with at least one family member with a history of mania or psychosis with onset within 6 weeks of childbirth (contributing 54 affected sibling pairs)	A genome scan with 494 microsatellite markers	Significant linkage with chromosome 16p13 Suggestive linkage with chromosome 8q24
Thippeswamy et al. (2017) [98]	101 healthy controls and 65 women with history of PP (mania, severe depression or psychosis within 6 weeks of delivery)	ESR1 (A/G rs9340799 and C/T rs2234693), HMCN1 (A/G rs2891230) and METTL13 (C/T rs2232825)	No significant associations between PP and polymorphisms of ERS1 or HMCN1. Significant association with T allele of METTL13 rs2232825 variant and PP
Middle et al. (2003) [99]	112 parous women with BD with a history of PP (mania or psychosis within 6 weeks of delivery), 50 parous women with BD without history of PP (*n* = 50), non-psychiatric comparison group (*n* = 110)	Oestrogen receptor alpha gene (ERα)	No significant association with PP
Coyle et al. (2000) [100]	97 women with BD with a history of PP (mania or psychosis within 14 days of delivery) and 72 controls (women attending general practitioner for non-psychiatric reasons matched for age and ethnicity)	Serotonin transporter gene (5-HTT; STin2.9, STin2.10 and STin2.12)	Significant association between STin2.12 and PP
Kumar et al. (2007) [101]	320 women (104 with PP [psychotic episode within 6 months of delivery], 102 with BD and 114 unaffected controls)	Serotonin receptor 2A (5-HT2A), serotonin receptor 2C (5-HT2C) and (5-HTT)	Significant association between PP and allele 10 of 5-HTTVNTR locus and with genotypic frequencies of the 5-HTTLPR
Weigelt et al. (2013) [102]	20 women with first onset PP, 20 healthy postpartum women and 20 healthy non-postpartum women	MicroRNAs	Significant downregulation of miR-146a expression in women with PP (compared to both control groups) Significant downregulation of miR-212 in women with PP and a history of BD
Dallaspezia (2011) [103]	17 parous women with BD with postpartum onset, 22 parous women with BD without a history of a postpartum mood episode and 28 men with BD (both control groups were matched for age at onset)	Polymorphisms of PER3 circadian rhythm clock gene	Significant association with PER3 and postpartum depressive onset of BD

PP: postpartum psychosis; BD: bipolar disorder.

**Table 3:** Key recommendations of review for further research.

Recommendations
Focus should be placed on further examining the nosology of postpartum psychosis to ensure these episodes can be adequately recorded and classified in clinical diagnostic criteria. Larger cohorts of women with postpartum psychosis are needed for future genome wide association studies. This may be achieved through international collaborative efforts and should comprise women with a history of narrowly defined postpartum psychosis (i.e., women with a history of mania or psychosis with onset soon after childbirth to reduce genetic heterogeneity). Prospective studies of pregnant women at high-risk are required to examine potential aetiological and triggering factors of postpartum psychosis. A range of methodological approaches should be utilised. Available data should be combined to develop predictive risk models of postpartum psychosis.

## Data Availability

No new data were created or analysed in this study. Data sharing is not applicable to this article.
